# Decima

**DOI:** 10.1186/2043-9113-5-S1-S11

**Published:** 2015-05-22

**Authors:** Anca Bucur

**Affiliations:** 1 High Tech Campus 11, 5656AE, Eindhoven, the Netherlands

## Characterisation

Service/tool, patient recruitment, restricted access.

## Description

To help enhance the participation of cancer patients in clinical trials we built an application that aims to support recruitment through the automatic evaluation of the eligibility of patients for trials based on matching the characteristics of the patient population required by the trial to the patient data available for instance in the hospital EHR (electronic health record).

The population to be enrolled in a trial is described by a set of free-text eligibility criteria that are both syntactically and semantically complex, which makes their automatic evaluation on the patient data in order to assess the eligibility of that patient for a set of trials a challenging task. The extraction and representation of the semantics of the trial criteria can be made more efficient by detecting structure and patterns in criteria, by information extraction and annotation techniques and by formalization of criteria.

**Figure 1:**
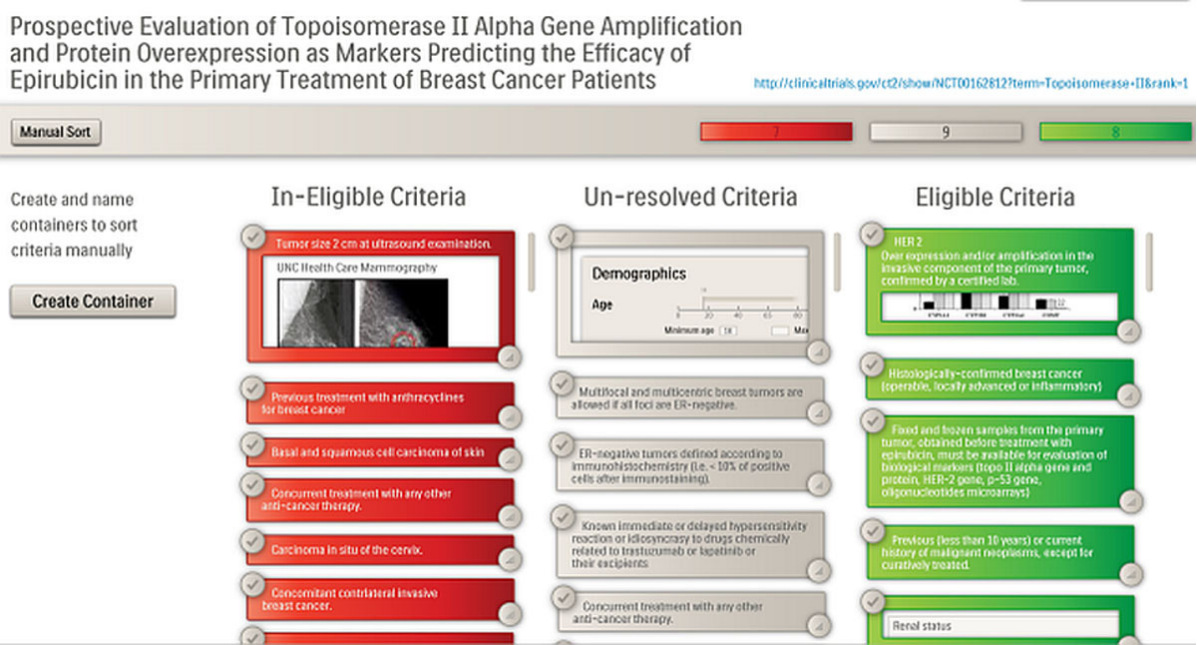
Detailed view of matched criteria for a patient and a clinical trial.

To automate the evaluation of eligibility of patients for trials, it is necessary to (1) extract and represent the semantics of the eligibility criteria in a machine-processable way and (2) to automatically match each criterion with the relevant data elements available for each patient. We address both aspects and propose a scalable, efficient and pragmatic application enabling automatic evaluation of eligibility of patients for a relevant set of clinical trials (Figure 1). The solution covers the flexible formalization of criteria and of other relevant trial metadata and the efficient management of these representations. Additionally, we rely on our standards-based semantic interoperability solution to provide shared semantics between formalized trial information and care data, and to enable automatic linkage and matching of trial criteria to patient data.

Our approach is generic and flexible, integrating loosely coupled components with well-defined standard interfaces and making use of prominent standards in the healthcare domain, such as HL7 RIM for our Clinical Data Model and BRIDG for the Trial Metadata Repository in which we preserve trial descriptions (e.g. eligibility criteria, associated formalisms and other relevant metadata). We combine the use of formal representations of eligibility criteria with a pragmatic and efficient implementation in which templates are defined, linked to execution logic, and extensively reused. The solution can integrate different formalisms, data representations and models to fit the needs of each new healthcare environment.

## Status of development

Version 3 (June 2014). Evaluation and validation at workshop in June 2014, several sessions at clinical sites in September and October 2014.

## Users

Oncologists, trial nurses, investigators, clinical researchers.

## Link

http://www.fp7-integrate.eu/index.php/downloads
